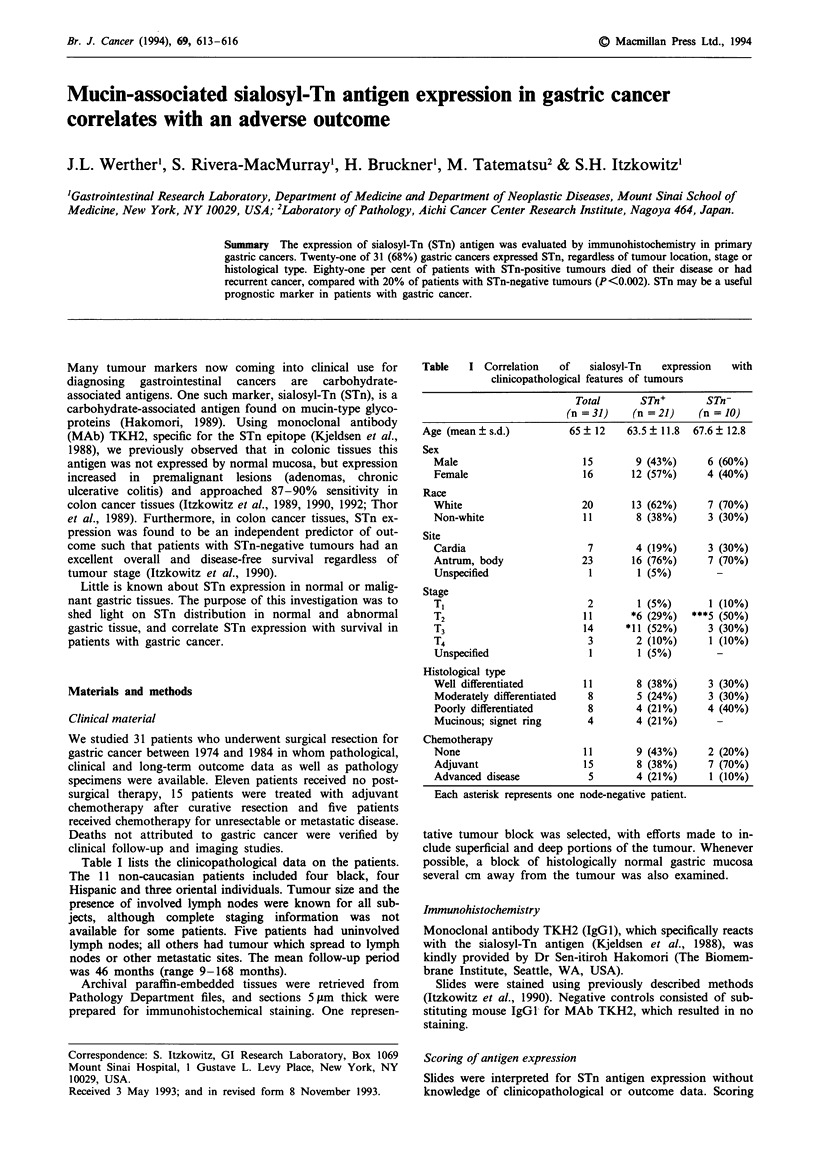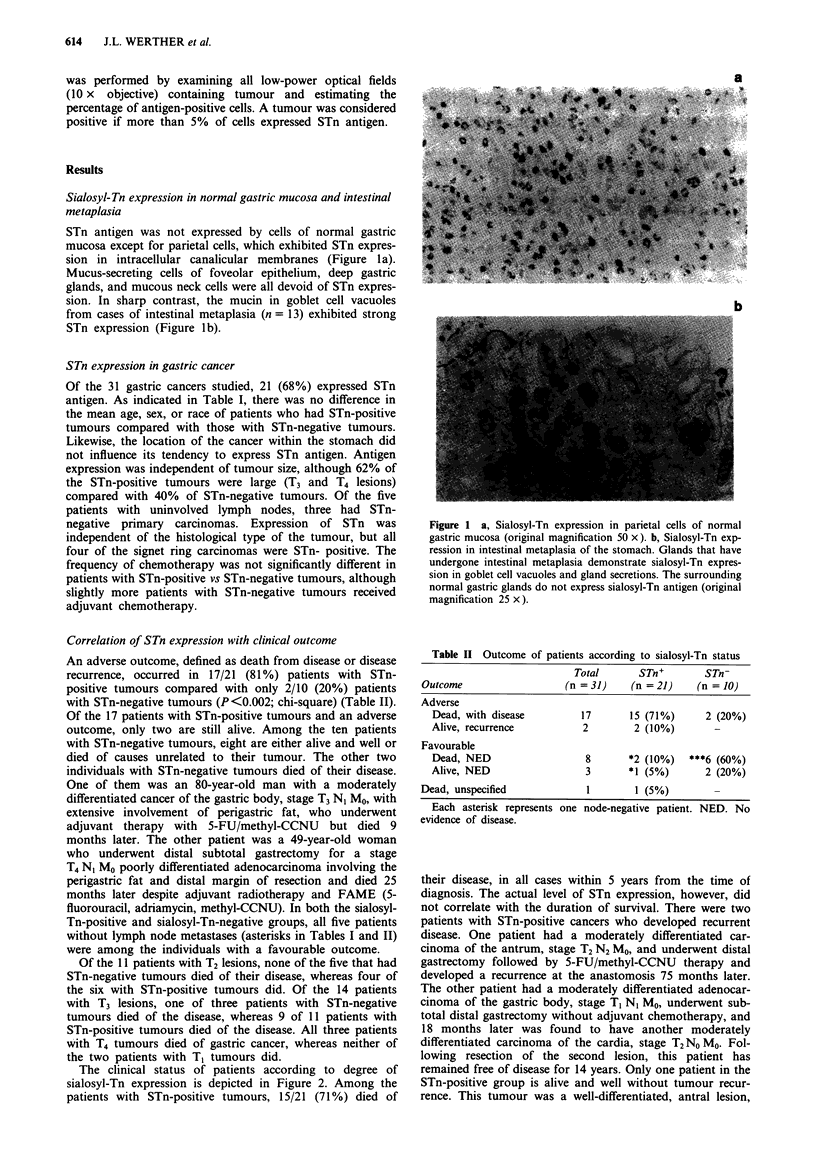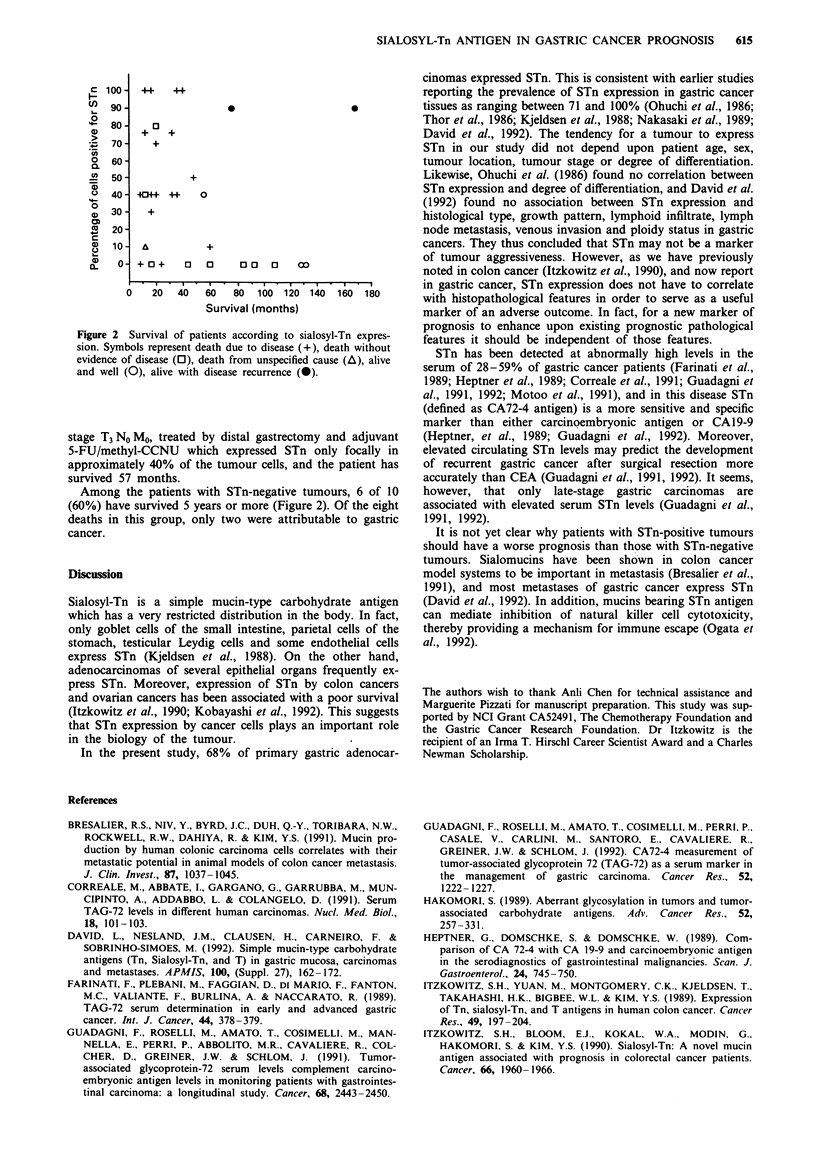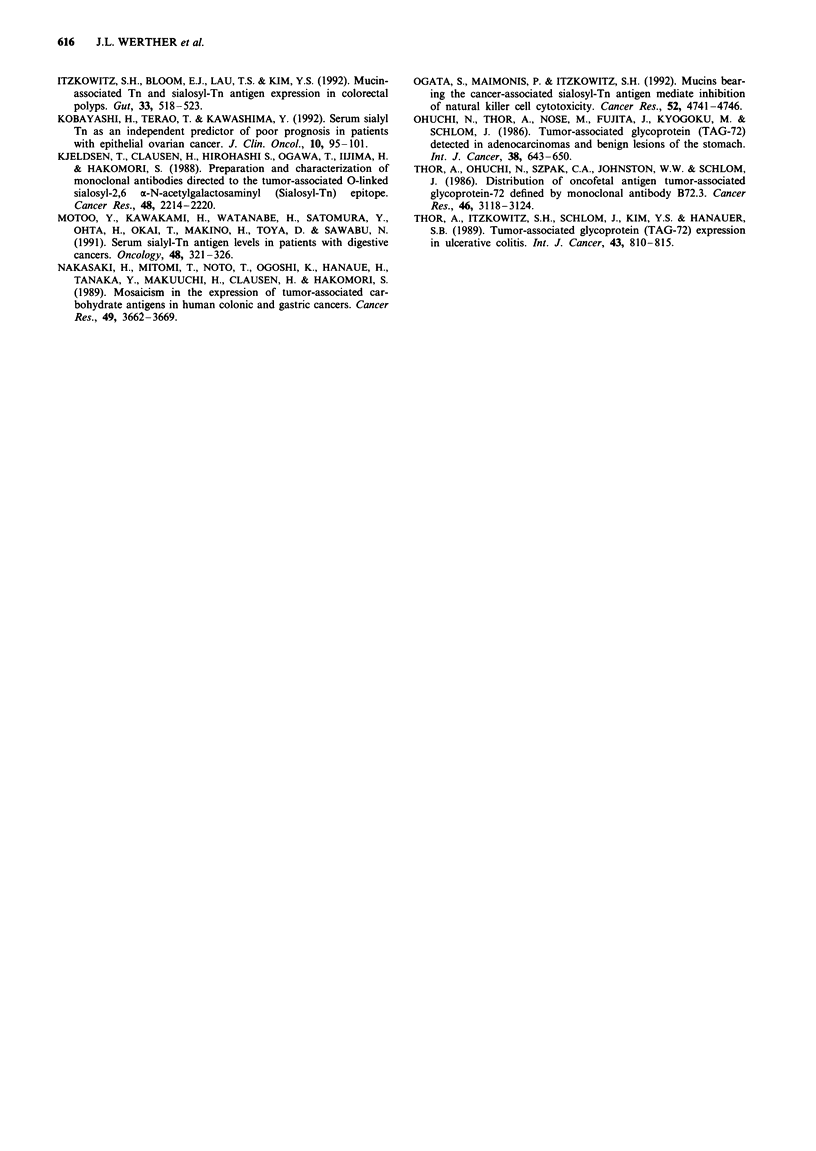# Mucin-associated sialosyl-Tn antigen expression in gastric cancer correlates with an adverse outcome.

**DOI:** 10.1038/bjc.1994.114

**Published:** 1994-03

**Authors:** J. L. Werther, S. Rivera-MacMurray, H. Bruckner, M. Tatematsu, S. H. Itzkowitz

**Affiliations:** Department of Medicine, Mount Sinai School of Medicine, New York, NY 10029.

## Abstract

**Images:**


Br. J. Cancer (1994), 69, 613-616 © Macmillan Press Ltd., 1994

### Mucin-associated sialosyl-Tn antigen expression in gastric cancer correlates with an adverse outcome

J.L. Werther[1], S. Rivera-MacMurray[1], H. Bruckner[1], M. Tatematsu[2] & S.H. Itzkowitz[1]

[1]*Gastrointestinal Research Laboratory, Department of Medicine and Department of Neoplastic Diseases, Mount Sinai School of Medicine, New York, NY 10029, USA;* [2]*Laboratory of Pathology, Aichi Cancer Center Research Institute, Nagoya 464, Japan.*

**Summary** The expression of sialosyl-Tn (STn) antigen was evaluated by immunohistochemistry in primary gastric cancers. Twenty-one of 31 (68%) gastric cancers expressed STn, regardless of tumour location, stage or histological type. Eighty-one per cent of patients with STn-positive tumours died of their disease or had recurrent cancer, compared with 20% of patients with STn-negative tumours ($P<0.002$). STn may be a useful prognostic marker in patients with gastric cancer.

Many tumour markers now coming into clinical use for diagnosing gastrointestinal cancers are carbohydrate-associated antigens. One such marker, sialosyl-Tn (STn), is a carbohydrate-associated antigen found on mucin-type glycoproteins (Hakomori, 1989). Using monoclonal antibody (MAb) TKH2, specific for the STn epitope (Kjeldsen *et al.*, 1988), we previously observed that in colonic tissues this antigen was not expressed by normal mucosa, but expression increased in premalignant lesions (adenomas, chronic ulcerative colitis) and approached 87–90% sensitivity in colon cancer tissues (Itzkowitz *et al.*, 1989, 1990, 1992; Thor *et al.*, 1989). Furthermore, in colon cancer tissues, STn expression was found to be an independent predictor of outcome such that patients with STn-negative tumours had an excellent overall and disease-free survival regardless of tumour stage (Itzkowitz *et al.*, 1990).

Little is known about STn expression in normal or malignant gastric tissues. The purpose of this investigation was to shed light on STn distribution in normal and abnormal gastric tissue, and correlate STn expression with survival in patients with gastric cancer.

### Materials and methods

### *Clinical material*

We studied 31 patients who underwent surgical resection for gastric cancer between 1974 and 1984 in whom pathological, clinical and long-term outcome data as well as pathology specimens were available. Eleven patients received no post-surgical therapy, 15 patients were treated with adjuvant chemotherapy after curative resection and five patients received chemotherapy for unresectable or metastatic disease. Deaths not attributed to gastric cancer were verified by clinical follow-up and imaging studies.

Table I lists the clinicopathological data on the patients. The 11 non-caucasian patients included four black, four Hispanic and three oriental individuals. Tumour size and the presence of involved lymph nodes were known for all subjects, although complete staging information was not available for some patients. Five patients had uninvolved lymph nodes; all others had tumour which spread to lymph nodes or other metastatic sites. The mean follow-up period was 46 months (range 9–168 months).

Archival paraffin-embedded tissues were retrieved from Pathology Department files, and sections 5 μm thick were prepared for immunohistochemical staining. One representative tumour block was selected, with efforts made to include superficial and deep portions of the tumour. Whenever possible, a block of histologically normal gastric mucosa several cm away from the tumour was also examined.

**Table I** Correlation of sialosyl-Tn expression with clinicopathological features of tumours

| | *Total* (n = *31)* | *STn*$^+$ (n = *21)* | *STn*$^-$ (n = *10)* |
|---|---|---|---|
| Age (mean ± s.d.) | 65 ± 12 | 63.5 ± 11.8 | 67.6 ± 12.8 |
| Sex | | | |
| Male | 15 | 9 (43%) | 6 (60%) |
| Female | 16 | 12 (57%) | 4 (40%) |
| Race | | | |
| White | 20 | 13 (62%) | 7 (70%) |
| Non-white | 11 | 8 (38%) | 3 (30%) |
| Site | | | |
| Cardia | 7 | 4 (19%) | 3 (30%) |
| Antrum, body | 23 | 16 (76%) | 7 (70%) |
| Unspecified | 1 | 1 (5%) | – |
| Stage | | | |
| $T_1$ | 2 | 1 (5%) | 1 (10%) |
| $T_2$ | 11 | *6 (29%) | ***5 (50%) |
| $T_3$ | 14 | *11 (52%) | 3 (30%) |
| $T_4$ | 3 | 2 (10%) | 1 (10%) |
| Unspecified | 1 | 1 (5%) | – |
| Histological type | | | |
| Well differentiated | 11 | 8 (38%) | 3 (30%) |
| Moderately differentiated | 8 | 5 (24%) | 3 (30%) |
| Poorly differentiated | 8 | 4 (21%) | 4 (40%) |
| Mucinous; signet ring | 4 | 4 (21%) | – |
| Chemotherapy | | | |
| None | 11 | 9 (43%) | 2 (20%) |
| Adjuvant | 15 | 8 (38%) | 7 (70%) |
| Advanced disease | 5 | 4 (21%) | 1 (10%) |

Each asterisk represents one node-negative patient.

### *Immunohistochemistry*

Monoclonal antibody TKH2 (IgG1), which specifically reacts with the sialosyl-Tn antigen (Kjeldsen *et al.*, 1988), was kindly provided by Dr Sen-itiroh Hakomori (The Biomembrane Institute, Seattle, WA, USA).

Slides were stained using previously described methods (Itzkowitz *et al.*, 1990). Negative controls consisted of substituting mouse IgG1 for MAb TKH2, which resulted in no staining.

### *Scoring of antigen expression*

Slides were interpreted for STn antigen expression without knowledge of clinicopathological or outcome data. Scoring

Correspondence: S. Itzkowitz, GI Research Laboratory, Box 1069 Mount Sinai Hospital, 1 Gustave L. Levy Place, New York, NY 10029, USA.
Received 3 May 1993; and in revised form 8 November 1993.

was performed by examining all low-power optical fields (10 × objective) containing tumour and estimating the percentage of antigen-positive cells. A tumour was considered positive if more than 5% of cells expressed STn antigen.

### Results

### *Sialosyl-Tn expression in normal gastric mucosa and intestinal metaplasia*

STn antigen was not expressed by cells of normal gastric mucosa except for parietal cells, which exhibited STn expression in intracellular canalicular membranes (Figure 1a). Mucus-secreting cells of foveolar epithelium, deep gastric glands, and mucous neck cells were all devoid of STn expression. In sharp contrast, the mucin in goblet cell vacuoles from cases of intestinal metaplasia ($n = 13$) exhibited strong STn expression (Figure 1b).

### *STn expression in gastric cancer*

Of the 31 gastric cancers studied, 21 (68%) expressed STn antigen. As indicated in Table I, there was no difference in the mean age, sex, or race of patients who had STn-positive tumours compared with those with STn-negative tumours. Likewise, the location of the cancer within the stomach did not influence its tendency to express STn antigen. Antigen expression was independent of tumour size, although 62% of the STn-positive tumours were large ($T_3$ and $T_4$ lesions) compared with 40% of STn-negative tumours. Of the five patients with uninvolved lymph nodes, three had STn-negative primary carcinomas. Expression of STn was independent of the histological type of the tumour, but all four of the signet ring carcinomas were STn- positive. The frequency of chemotherapy was not significantly different in patients with STn-positive *vs* STn-negative tumours, although slightly more patients with STn-negative tumours received adjuvant chemotherapy.

### *Correlation of STn expression with clinical outcome*

An adverse outcome, defined as death from disease or disease recurrence, occurred in 17/21 (81%) patients with STn-positive tumours compared with only 2/10 (20%) patients with STn-negative tumours ($P < 0.002$; chi-square) (Table II). Of the 17 patients with STn-positive tumours and an adverse outcome, only two are still alive. Among the ten patients with STn-negative tumours, eight are either alive and well or died of causes unrelated to their tumour. The other two individuals with STn-negative tumours died of their disease. One of them was an 80-year-old man with a moderately differentiated cancer of the gastric body, stage $T_3 N_1 M_0$, with extensive involvement of perigastric fat, who underwent adjuvant therapy with 5-FU/methyl-CCNU but died 9 months later. The other patient was a 49-year-old woman who underwent distal subtotal gastrectomy for a stage $T_4 N_1 M_0$ poorly differentiated adenocarcinoma involving the perigastric fat and distal margin of resection and died 25 months later despite adjuvant radiotherapy and FAME (5-fluorouracil, adriamycin, methyl-CCNU). In both the sialosyl-Tn-positive and sialosyl-Tn-negative groups, all five patients without lymph node metastases (asterisks in Tables I and II) were among the individuals with a favourable outcome.

Of the 11 patients with $T_2$ lesions, none of the five that had STn-negative tumours died of their disease, whereas four of the six with STn-positive tumours did. Of the 14 patients with $T_3$ lesions, one of three patients with STn-negative tumours died of the disease, whereas 9 of 11 patients with STn-positive tumours died of the disease. All three patients with $T_4$ tumours died of gastric cancer, whereas neither of the two patients with $T_1$ tumours did.

The clinical status of patients according to degree of sialosyl-Tn expression is depicted in Figure 2. Among the patients with STn-positive tumours, 15/21 (71%) died of their disease, in all cases within 5 years from the time of diagnosis. The actual level of STn expression, however, did not correlate with the duration of survival. There were two patients with STn-positive cancers who developed recurrent disease. One patient had a moderately differentiated carcinoma of the antrum, stage $T_2 N_2 M_0$, and underwent distal gastrectomy followed by 5-FU/methyl-CCNU therapy and developed a recurrence at the anastomosis 75 months later. The other patient had a moderately differentiated adenocarcinoma of the gastric body, stage $T_1 N_1 M_0$, underwent subtotal distal gastrectomy without adjuvant chemotherapy, and 18 months later was found to have another moderately differentiated carcinoma of the cardia, stage $T_2 N_0 M_0$. Following resection of the second lesion, this patient has remained free of disease for 14 years. Only one patient in the STn-positive group is alive and well without tumour recurrence. This tumour was a well-differentiated, antral lesion,

**Figure 1** **a**, Sialosyl-Tn expression in parietal cells of normal gastric mucosa (original magnification 50 ×). **b**, Sialosyl-Tn expression in intestinal metaplasia of the stomach. Glands that have undergone intestinal metaplasia demonstrate sialosyl-Tn expression in goblet cell vacuoles and gland secretions. The surrounding normal gastric glands do not express sialosyl-Tn antigen (original magnification 25 ×).

**Table II** Outcome of patients according to sialosyl-Tn status

| *Outcome* | *Total* (n = *31*) | *STn*$^+$ (n = *21*) | *STn*$^-$ (n = *10*) |
|---|---|---|---|
| Adverse | | | |
| Dead, with disease | 17 | 15 (71%) | 2 (20%) |
| Alive, recurrence | 2 | 2 (10%) | – |
| Favourable | | | |
| Dead, NED | 8 | *2 (10%) | ***6 (60%) |
| Alive, NED | 3 | *1 (5%) | 2 (20%) |
| Dead, unspecified | 1 | 1 (5%) | – |

Each asterisk represents one node-negative patient. NED. No evidence of disease.

**Figure 2** Survival of patients according to sialosyl-Tn expression. Symbols represent death due to disease (+), death without evidence of disease (□), death from unspecified cause (Δ), alive and well (○), alive with disease recurrence (●).

stage $T_3 N_0 M_0$, treated by distal gastrectomy and adjuvant 5-FU/methyl-CCNU which expressed STn only focally in approximately 40% of the tumour cells, and the patient has survived 57 months.

Among the patients with STn-negative tumours, 6 of 10 (60%) have survived 5 years or more (Figure 2). Of the eight deaths in this group, only two were attributable to gastric cancer.

### Discussion

Sialosyl-Tn is a simple mucin-type carbohydrate antigen which has a very restricted distribution in the body. In fact, only goblet cells of the small intestine, parietal cells of the stomach, testicular Leydig cells and some endothelial cells express STn (Kjeldsen *et al.*, 1988). On the other hand, adenocarcinomas of several epithelial organs frequently express STn. Moreover, expression of STn by colon cancers and ovarian cancers has been associated with a poor survival (Itzkowitz *et al.*, 1990; Kobayashi *et al.*, 1992). This suggests that STn expression by cancer cells plays an important role in the biology of the tumour.

In the present study, 68% of primary gastric adenocarcinomas expressed STn. This is consistent with earlier studies reporting the prevalence of STn expression in gastric cancer tissues as ranging between 71 and 100% (Ohuchi *et al.*, 1986; Thor *et al.*, 1986; Kjeldsen *et al.*, 1988; Nakasaki *et al.*, 1989; David *et al.*, 1992). The tendency for a tumour to express STn in our study did not depend upon patient age, sex, tumour location, tumour stage or degree of differentiation. Likewise, Ohuchi *et al.* (1986) found no correlation between STn expression and degree of differentiation, and David *et al.* (1992) found no association between STn expression and histological type, growth pattern, lymphoid infiltrate, lymph node metastasis, venous invasion and ploidy status in gastric cancers. They thus concluded that STn may not be a marker of tumour aggressiveness. However, as we have previously noted in colon cancer (Itzkowitz *et al.*, 1990), and now report in gastric cancer, STn expression does not have to correlate with histopathological features in order to serve as a useful marker of an adverse outcome. In fact, for a new marker of prognosis to enhance upon existing prognostic pathological features it should be independent of those features.

STn has been detected at abnormally high levels in the serum of 28–59% of gastric cancer patients (Farinati *et al.*, 1989; Heptner *et al.*, 1989; Correale *et al.*, 1991; Guadagni *et al.*, 1991, 1992; Motoo *et al.*, 1991), and in this disease STn (defined as CA72-4 antigen) is a more sensitive and specific marker than either carcinoembryonic antigen or CA19-9 (Heptner, *et al.*, 1989; Guadagni *et al.*, 1992). Moreover, elevated circulating STn levels may predict the development of recurrent gastric cancer after surgical resection more accurately than CEA (Guadagni *et al.*, 1991, 1992). It seems, however, that only late-stage gastric carcinomas are associated with elevated serum STn levels (Guadagni *et al.*, 1991, 1992).

It is not yet clear why patients with STn-positive tumours should have a worse prognosis than those with STn-negative tumours. Sialomucins have been shown in colon cancer model systems to be important in metastasis (Bresalier *et al.*, 1991), and most metastases of gastric cancer express STn (David *et al.*, 1992). In addition, mucins bearing STn antigen can mediate inhibition of natural killer cell cytotoxicity, thereby providing a mechanism for immune escape (Ogata *et al.*, 1992).

The authors wish to thank Anli Chen for technical assistance and Marguerite Pizzati for manuscript preparation. This study was supported by NCI Grant CA52491, The Chemotherapy Foundation and the Gastric Cancer Research Foundation. Dr Itzkowitz is the recipient of an Irma T. Hirschl Career Scientist Award and a Charles Newman Scholarship.

### References

BRESALIER, R.S., NIV, Y., BYRD, J.C., DUH, Q.-Y., TORIBARA, N.W., ROCKWELL, R.W., DAHIYA, R. & KIM, Y.S. (1991). Mucin production by human colonic carcinoma cells correlates with their metastatic potential in animal models of colon cancer metastasis. *J. Clin. Invest.*, **87**, 1037–1045.

CORREALE, M., ABBATE, I., GARGANO, G., GARRUBBA, M., MUNCIPINTO, A., ADDABBO, L. & COLANGELO, D. (1991). Serum TAG-72 levels in different human carcinomas. *Nucl. Med. Biol.*, **18**, 101–103.

DAVID, L., NESLAND, J.M., CLAUSEN, H., CARNEIRO, F. & SOBRINHO-SIMOES, M. (1992). Simple mucin-type carbohydrate antigens (Tn, Sialosyl-Tn, and T) in gastric mucosa, carcinomas and metastases. *APMIS*, **100**, (Suppl. 27), 162–172.

FARINATI, F., PLEBANI, M., FAGGIAN, D., DI MARIO, F., FANTON, M.C., VALIANTE, F., BURLINA, A. & NACCARATO, R. (1989). TAG-72 serum determination in early and advanced gastric cancer. *Int. J. Cancer*, **44**, 378–379.

GUADAGNI, F., ROSELLI, M., AMATO, T., COSIMELLI, M., MANNELLA, E., PERRI, P., ABBOLITO, M.R., CAVALIERE, R., COLCHER, D., GREINER, J.W. & SCHLOM, J. (1991). Tumor-associated glycoprotein-72 serum levels complement carcinoembryonic antigen levels in monitoring patients with gastrointestinal carcinoma: a longitudinal study. *Cancer*, **68**, 2443–2450.

GUADAGNI, F., ROSELLI, M., AMATO, T., COSIMELLI, M., PERRI, P., CASALE, V., CARLINI, M., SANTORO, E., CAVALIERE, R., GREINER, J.W. & SCHLOM, J. (1992). CA72-4 measurement of tumor-associated glycoprotein 72 (TAG-72) as a serum marker in the management of gastric carcinoma. *Cancer Res.*, **52**, 1222–1227.

HAKOMORI, S. (1989). Aberrant glycosylation in tumors and tumor-associated carbohydrate antigens. *Adv. Cancer Res.*, **52**, 257–331.

HEPTNER, G., DOMSCHKE, S. & DOMSCHKE, W. (1989). Comparison of CA 72-4 with CA 19-9 and carcinoembryonic antigen in the serodiagnostics of gastrointestinal malignancies. *Scan. J. Gastroenterol.*, **24**, 745–750.

ITZKOWITZ, S.H., YUAN, M., MONTGOMERY, C.K., KJELDSEN, T., TAKAHASHI, H.K., BIGBEE, W.L. & KIM, Y.S. (1989). Expression of Tn, sialosyl-Tn, and T antigens in human colon cancer. *Cancer Res.*, **49**, 197–204.

ITZKOWITZ, S.H., BLOOM, E.J., KOKAL, W.A., MODIN, G., HAKOMORI, S. & KIM, Y.S. (1990). Sialosyl-Tn: A novel mucin antigen associated with prognosis in colorectal cancer patients. *Cancer*, **66**, 1960–1966.

ITZKOWITZ, S.H., BLOOM, E.J., LAU, T.S. & KIM, Y.S. (1992). Mucin-associated Tn and sialosyl-Tn antigen expression in colorectal polyps. *Gut*, **33**, 518–523.

KOBAYASHI, H., TERAO, T. & KAWASHIMA, Y. (1992). Serum sialyl Tn as an independent predictor of poor prognosis in patients with epithelial ovarian cancer. *J. Clin. Oncol.*, **10**, 95–101.

KJELDSEN, T., CLAUSEN, H., HIROHASHI S., OGAWA, T., IIJIMA, H. & HAKOMORI, S. (1988). Preparation and characterization of monoclonal antibodies directed to the tumor-associated O-linked sialosyl-2,6 α-N-acetylgalactosaminyl (Sialosyl-Tn) epitope. *Cancer Res.*, **48**, 2214–2220.

MOTOO, Y., KAWAKAMI, H., WATANABE, H., SATOMURA, Y., OHTA, H., OKAI, T., MAKINO, H., TOYA, D. & SAWABU, N. (1991). Serum sialyl-Tn antigen levels in patients with digestive cancers. *Oncology*, **48**, 321–326.

NAKASAKI, H., MITOMI, T., NOTO, T., OGOSHI, K., HANAUE, H., TANAKA, Y., MAKUUCHI, H., CLAUSEN, H. & HAKOMORI, S. (1989). Mosaicism in the expression of tumor-associated carbohydrate antigens in human colonic and gastric cancers. *Cancer Res.*, **49**, 3662–3669.

OGATA, S., MAIMONIS, P. & ITZKOWITZ, S.H. (1992). Mucins bearing the cancer-associated sialosyl-Tn antigen mediate inhibition of natural killer cell cytotoxicity. *Cancer Res.*, **52**, 4741–4746.

OHUCHI, N., THOR, A., NOSE, M., FUJITA, J., KYOGOKU, M. & SCHLOM, J. (1986). Tumor-associated glycoprotein (TAG-72) detected in adenocarcinomas and benign lesions of the stomach. *Int. J. Cancer*, **38**, 643–650.

THOR, A., OHUCHI, N., SZPAK, C.A., JOHNSTON, W.W. & SCHLOM, J. (1986). Distribution of oncofetal antigen tumor-associated glycoprotein-72 defined by monoclonal antibody B72.3. *Cancer Res.*, **46**, 3118–3124.

THOR, A., ITZKOWITZ, S.H., SCHLOM, J., KIM, Y.S. & HANAUER, S.B. (1989). Tumor-associated glycoprotein (TAG-72) expression in ulcerative colitis. *Int. J. Cancer*, **43**, 810–815.